# Supplementary material for: Resilience of the topological phases to frustration
Source: Sci Rep. 2021 Mar 22;11:6508. doi: 10.1038/s41598-021-86009-4 (PMC7985368; doi:10.1038/s41598-021-86009-4)
Supplement: Supplementary file 1 — Supplementary Information [file 41598_2021_86009_MOESM1_ESM.pdf]

Resilience of the Topological Phases to frustration

Vanja Marić[1,2], Fabio Franchini[1], Domagoj Kuić[1], and Salvatore Marco Giampaolo[1]

[1]Division of Theoretical Physics, Rudjer Bošković Institute, Bijenička cesta 54, 10000 Zagreb, Croatia
[2]SISSA and INFN, via Bonomea 265, 34136 Trieste, Italy

ABSTRACT

This is the supplementary information for the paper Resilience of the Topological Phases to frustration. Here we diagonalize the Cluster-Ising chain and Kitaev chain, and compute the order parameter.

RBI-ThPhys-2020-20

1 Cluster-Ising Chain

1.1 Diagonalization

The Cluster-Ising chain Hamiltonian

$$H = \cos\phi \sum_{j=1}^{N} \sigma_j^x \sigma_{j+1}^x + \sin\phi \sum_{j=1}^{N} \sigma_{j-1}^y \sigma_j^z \sigma_{j+1}^y \tag{1}$$

in terms of Jordan-Wigner fermions

$$c_j = \Big(\bigotimes_{l=1}^{j-1} \sigma_l^z\Big) \frac{\sigma_j^x + \imath\sigma_j^y}{2}, \quad c_j^\dagger = \Big(\bigotimes_{l=1}^{j-1} \sigma_l^z\Big) \frac{\sigma_j^x - \imath\sigma_j^y}{2}, \tag{2}$$

reads

$$\begin{aligned} H = &-\cos\phi \left[\sum_{j=1}^{N-1} (c_j c_{j+1} + c_j c_{j+1}^\dagger) - \Pi^z (c_N c_1 + c_N c_1^\dagger) + \text{h.c.} \right] \\ &+ \sin\phi \left[\sum_{j=2}^{N-1} (c_{j-1} c_{j+1} - c_{j-1} c_{j+1}^\dagger) - \Pi^z (c_{N-1} c_1 + c_N c_2 - c_{N-1} c_1^\dagger - c_N c_2^\dagger) + \text{h.c.} \right]. \end{aligned} \tag{3}$$

Because of the presence of Π^z, the Hamiltonian is not quadratic in the fermions, but becomes such in each Π^z parity sector. Namely, we can split the Hamiltonian as

$$H = \frac{1+\Pi^z}{2} H^+ \frac{1+\Pi^z}{2} + \frac{1-\Pi_z}{2} H^- \frac{1-\Pi_z}{2}\ , \tag{4}$$

where both H^+ and H^- are quadratic. As such, they can be brought to a form of free fermions.

This is achieved by first writing $H^\pm$ in terms of the Fourier transformed Jordan-Wigner fermions,

$$b_q = \frac{1}{\sqrt{N}} \sum_{j=1}^{N} c_j\, e^{-\imath q j}, \quad b_q^\dagger = \frac{1}{\sqrt{N}} \sum_{j=1}^{N} c_j^\dagger\, e^{\imath q j}, \tag{5}$$

for $q \in \Gamma^\pm$, where the two sets of momenta are given by $\Gamma^- = \{2\pi k/N\}$ and $\Gamma^+ = \{2\pi(k+\frac{1}{2})/N\}$ with k running over all integers between 0 and $N-1$. Then the Bogoliubov rotation

$$\begin{aligned} &a_q = \cos\theta_q\, b_q + \imath \sin\theta_q\, b_{-q}^\dagger, \quad q \neq 0,\pi \\ &a_q = b_q, \quad q = 0,\pi \end{aligned} \tag{6}$$

with the Bogoliubov angle

$$\theta_q = \arctan \frac{|\sin\phi + \cos\phi\ e^{\imath 3q}| + \cos\phi \cos q - \sin\phi \cos 2q}{\cos\phi \sin q - \sin\phi \sin 2q} \tag{7}$$

brings $H^{\pm}$ to a free fermionic form. We end up with

$$H^{\pm} = \sum_{q\in\Gamma^{\pm}} \varepsilon_q \left(a_q^{\dagger}a_q - \frac{1}{2}\right), \tag{8}$$

where the energies are given by

$$\begin{aligned} \varepsilon_q &= 2\sqrt{1+\sin 2\phi \cos 3q} && \forall q \neq 0,\pi, \\ \varepsilon_0 &= 2(\sin\phi + \cos\phi) && q = 0 \in \Gamma^{-}, \\ \varepsilon_\pi &= 2(\sin\phi - \cos\phi) && q = \pi \in \Gamma^{+}. \end{aligned} \tag{9}$$

The eigenstates of H are formed starting from the vacuum states $|0^{\pm}\rangle$, which satisfy $a_q|0^{\pm}\rangle = 0$ for $q\in\Gamma^{\pm}$, and applying Bogoliubov fermions creation operators, while taking care of the parity requirements in (4). The vacuum states are given by

$$|0^{\pm}\rangle = \prod_{0<q<\pi,\, q\in\Gamma^{\pm}} \left(\cos\theta_q - \imath \sin\theta_q\, b_q^{\dagger} b_{-q}^{\dagger}\right)|0\rangle, \tag{10}$$

where $|0\rangle$ is the vacuum for Jordan-Wigner fermions, satisfying $c_j|0\rangle = 0$. In particular, $|0\rangle = |\uparrow\uparrow\ldots\uparrow\rangle$ is the state of all spin up. The vacuum states $|0^{+}\rangle$ and $|0^{-}\rangle$ both have parity $\Pi^z = +1$ by construction. The parity requirements in (4) imply that the eigenstates of H belonging to $\Pi^z = -1$ sector are of the form $a_{q_1}^{\dagger}a_{q_2}^{\dagger}\ldots a_{q_m}^{\dagger}|0^{-}\rangle$ with $q_i\in\Gamma^{-}$ and m odd, while $\Pi^z = +1$ sector eigenstates are of the same form but with $q_i\in\Gamma^{+}$, m even and the vacuum $|0^{+}\rangle$ used. The ground states are given explicitly in the main text.

Let us also note one technical subtlety of the model. The Bogoliubov angle θ_q, defined by (7) can become undefined for some modes $q\neq 0,\pi$ also point-wise, by fine–tuning of the parameters N and ϕ. The Bogoliubov angle for these modes θ_q can be defined in the same way as for modes $q = 0,\pi$ in the next section and the problem with them can be circumvented. These points do not have different expectation values of observables and can be neglected.

1.2 Majorana correlators

We are going to present the computation of two-point correlators of Majorana fermions

$$A_j = c_j^{\dagger} + c_j, \quad B_j = \imath(c_j^{\dagger} - c_j), \tag{11}$$

in the cluster phase in some details, because essentially the same reasoning is valid also for the Kitaev chain. For this computation it is convenient to write the Hamiltonians $H^{\pm}$ in terms of positive energy fermions d_q, that we now define. For $q\neq 0,\pi$ we put simply

$$d_q = a_q. \tag{12}$$

For the modes $q = 0,\pi$ the Bogoliubov angle (7) is undefined. We are going to define it also for these modes and use the analogoue of (6) to define d_q. First, we note that the Bogoliubov angle defined by (7) for $q\neq 0,\pi$ satisfies

$$e^{\imath 2\theta_q} = e^{-\imath 2q}\frac{\sin\phi + \cos\phi\; e^{\imath 3q}}{|\sin\phi + \cos\phi\; e^{\imath 3q}|}. \tag{13}$$

Although for modes $q = 0,\pi$ the expression (7) is undefined, there is no problems with expression (13). We exploit this property and define

$$\theta_q \equiv \frac{1}{2\imath}\log e^{\imath 2\theta_q}, \quad q = 0,\pi, \tag{14}$$

where by $e^{\imath 2\theta_q}$ the expression on the right hand side of (13) is understood. Having θ_q we define, as in (6),

$$d_q = \cos\theta_q\, b_q + i\sin\theta_q\, b_{-q}^{\dagger}, \quad q = 0,\pi. \tag{15}$$

Since for $q = 0,\pi$ we have

$$e^{\imath 2\theta_q} = \mathrm{sgn}(\varepsilon_q) \tag{16}$$

these definitions will result in the property that all fermions d_q have positive energies, i.e. we can write

$$H^{\pm} = \sum_{q\in\Gamma^{\pm}} |\varepsilon_q| \left(d_q^{\dagger}d_q - \frac{1}{2}\right). \tag{17}$$

With these definitions the ground state of H^- (H^+), let's denote it by $|g,H^-\rangle$ ($|g,H^+\rangle$) is the state that is annihilated by all d_q for $q\in\Gamma^-$ (Γ^+), i.e. $d_q|g,H^-\rangle=0$.

It is easy to see from the exact solution that the ground state $|g\rangle$ of the Cluster-Ising Hamiltonian H, coincides with $|g,H^+\rangle$ for $\phi\in(\pi/4,3\pi/4)$ and with $|g,H^-\rangle$ for $\phi\in(-3\pi/4,-\pi/4)$. We note that a typical effect of geometrical frustration[?,?,?,?], which is not the case here, is that $|g\rangle$ does not coincide with either of them, because of the parity requirements in (4).

Let us thus compute the Majorana correlation functions in the state $|g,H^-\rangle$, identical analysis can be made also for $|g,H^+\rangle$. From the definitions (12) and (15), we obtain

$$b_q=\cos\theta_q d_q+\imath\sin\theta_q d_{-q}^\dagger. \tag{18}$$

Now, using the definition (5) we get

$$c_j=\frac{1}{\sqrt{N}}\sum_{q\in\Gamma^-}(\cos\theta_q d_q-\imath\sin\theta_q d_{-q}^\dagger)e^{\imath qj}, \tag{19}$$

from which we get easily the correlation functions

$$\langle c_jc_l\rangle_{g,H^-}=\frac{\imath}{2N}\sum_{q\in\Gamma^-}\sin2\theta_q e^{\imath q(j-l)}, \tag{20}$$

$$\langle c_jc_l^\dagger\rangle_{g,H^-}=\frac{1}{2N}\sum_{q\in\Gamma^-}(1+\cos2\theta_q)e^{\imath q(j-l)}, \tag{21}$$

Finally, from the definition (11) of Majorana fermions we get

$$\langle A_jA_l\rangle_{g,H^-}=\langle B_jB_l\rangle_{g,H^-}=\delta_{jl}, \tag{22}$$

$$-\imath\langle A_jB_l\rangle_{g,H^-}=\frac{1}{N}\sum_{q\in\Gamma^-}e^{i2\theta_q}e^{-iq(j-l)}. \tag{23}$$

The only difference in the ground state $|g,H^+\rangle$ is that the sum in (23) would be over Γ^+ instead of Γ^-. In the limit of a large system the results are the same since the sum becomes an integral, exponentially fast. We have

$$\langle A_jA_l\rangle_{g,H^\pm}=\langle B_jB_l\rangle_{g,H^\pm}=\delta_{jl}, \tag{24}$$

$$-\imath\langle A_jB_l\rangle_{g,H^\pm}\overset{N\to\infty}{\simeq}\int_0^{2\pi}e^{i2\theta_q}e^{-iq(j-l)}\frac{dq}{2\pi}. \tag{25}$$

In the antiferromagnetic phase the ground state of the Cluster-Ising chain with FBCs is not anymore a vacuum state for positive energy fermions, i.e. the ground state coincides neither with the ground state of H^+ nor with the one of H^-. Instead, it corresponds to the vacuum state with one excitation on top of it. Correspondingly, the Majorana correlation functions acquire corrections of order $1/N$. For the ground state for $\phi\in(-\pi/4,0)$ when N is divisible by 3, presented in the main text, and given by

$$|g\rangle=(u_1a_0^\dagger+u_2a_{\frac{2\pi}{3}}^\dagger+u_3a_{-\frac{2\pi}{3}}^\dagger)\,|0^-\rangle\,. \tag{26}$$

After some algebra we get

$$\begin{aligned}
\langle A_jA_l\rangle_g &= \delta_{jl}-\frac{2\imath}{N}(|u_2|^2-|u_3|^2)\sin\left[\frac{2\pi}{3}(j-l)\right]-\frac{2\imath}{N}\left[(u_1^*u_2-u_3^*u_1)e^{\imath\frac{\pi}{3}(j+l-1)}+\text{c.c.}\right]\sin\left[\frac{\pi}{3}(j-l)\right];\\
\langle B_jB_l\rangle_g &= \delta_{jl}-\frac{2\imath}{N}(|u_2|^2-|u_3|^2)\sin\left[\frac{2\pi}{3}(j-l)\right]-\frac{2\imath}{N}\left[(u_1^*u_2-u_3^*u_1)e^{\imath\frac{\pi}{3}(j+l+1)}+\text{c.c.}\right]\sin\left[\frac{\pi}{3}(j-l)\right];\\
-\imath\langle A_jB_l\rangle_g &\overset{N\to\infty}{\simeq} \int_0^{2\pi}\frac{\cos\phi+\sin\phi\; e^{-\imath3q}}{|\cos\phi+\sin\phi\; e^{-\imath3q}|}e^{-\imath q(j-l-1)}\frac{dq}{2\pi}-\frac{2}{N}\left\{|u_1|^2+(|u_2|^2+|u_3|^2)\cos\left[\frac{2\pi}{3}(j-l-1)\right]\right\}\\
&\quad -\frac{2}{N}\left[(u_1^*u_2+u_3^*u_1)e^{\imath\frac{\pi}{3}(j+l)}+\text{c.c.}\right]\cos\left[\frac{\pi}{3}(j-l-1)\right]-\frac{2}{N}\left[u_2^*u_3\;e^{-\imath\frac{2\pi}{3}(j+l)}+\text{c.c.}\right],
\end{aligned} \tag{27}$$

The ground state and the correlators when N is not divisible by 3 can be reproduced from these expressions by taking formally $u_2=u_3=0$.

1.3 Spin-correlation functions

In this section we compute the spin-correlation functions $\langle\sigma_1^x\sigma_{1+r}^x\rangle_g$ in the ground state $|g\rangle$ in the antiferromagnetic phase of the model, given in the main text. We start from the relation

$$\sigma_1^x\sigma_{1+r}^x = (-1)^r\prod_{j=1}^{r}(-iA_{j+1}B_j) \tag{28}$$

and use the Wick theorem to reduce the spin-correlation functions to the pfaffian of the Majorana correlation matrix.

Let us first discuss the applicability of the Wick theorem. When N is not divisible by 3, or when N is divisible by 3 and $u_j = 1$ for some $j \in \{1,2,3\}$, it's easy to write the ground state as a vacuum state for some fermionic operators, so the Wick theorem can be applied. In a more general case when N is divisible by 3 it's a bit more complicated. If some coefficient u_j is equal to zero then the same argument as in ref.[?] can be given for the applicability. If all of them are non-zero we proceed in the following way. First, similarly to ref.[?], we define the fermions α_q by

$$\alpha_p = \frac{1}{(|u_2|^2+|u_3|^2)^{1/2}}\left(u_2a_p^\dagger + u_3a_{-p}^\dagger\right), \quad \alpha_{-p} = \frac{1}{(|u_2|^2+|u_3|^2)^{1/2}}\left(u_3a_p - u_2a_{-p}\right), \tag{29}$$

for $p \equiv 2\pi/3$, and by $\alpha_q = a_q$ for $q \neq p, -p$. Then we make another similar step and define the fermions β_q by

$$\beta_0 = \alpha_{-p}, \quad \beta_p = u_1a_0^\dagger + (|u_2|^2+|u_3|^2)^{1/2}\alpha_p, \quad \beta_{-p} = (|u_2|^2+|u_3|^2)^{1/2}a_0 - u_1\alpha_p^\dagger,$$

and by $\beta_q = \alpha_q$ for $q \neq 0, p, -p$. Then the state (26) satisfies $|g\rangle = \beta_p|0^-\rangle$ and it's easy to see that it is annihilated by all β_q, i.e. we have $\beta_q|g\rangle = 0$ for all $q \in \Gamma^-$. Thus, we have expressed the ground state as the vacuum for fermions β_q. Moreover, since Majorana fermions A_j, B_j can be expressed as a linear combination of fermions $a_q, a_q^\dagger$, they can also be expressed as a linear combination of $\beta_q, \beta_q^\dagger$. Therefore, Wick theorem can be applied.

Applying the Wick theorem, we express the spin-correlation function as a pfaffian

$$\langle\sigma_1^x\sigma_{1+r}^x\rangle_g = (-1)^{r+\lfloor\frac{r}{2}\rfloor}\,\mathrm{pf}\begin{pmatrix}\mathbf{A} & \mathbf{C}\\ -\mathbf{C}^\mathrm{T} & -\mathbf{B}\end{pmatrix}. \tag{30}$$

Here $\mathbf{A}$ and $\mathbf{B}$ are antisymmetric $r\times r$ matrices, defined by the elements $\mathbf{A}_{j,l} = \langle A_{j+1}A_{l+1}\rangle_g$ and $\mathbf{B}_{j,l} = \langle B_jB_l\rangle_g$ for $j<l$, while $\mathbf{C}$ is an $r\times r$ matrix with the elements $\mathbf{C}_{j,l} = -\imath\langle A_{j+1}B_l\rangle_g$ (j and l range from 1 to r). In a more simple special case when the correlators $\langle A_jA_l\rangle_g$ and $\langle B_jB_l\rangle_g$ in the ground state $|g\rangle$ are zero for $j\neq l$, the spin correlations become simply the determinant

$$\langle\sigma_1^x\sigma_{1+r}^x\rangle_g = (-1)^r\det\mathbf{C}, \tag{31}$$

as in ref.[?].

Now let us compute the spin-correlation functions. When N is not divisible by 3 the correlators $\langle A_jA_l\rangle_g$ and $\langle B_jB_l\rangle_g$ are zero so we can use (31). Approximating the sum in (27) by integral we get

$$\mathbf{C}_{j,l} \overset{N\to\infty}{\simeq} \int_0^{2\pi}\frac{1+\tan\phi\; e^{-\imath 3q}}{|1+\tan\phi\; e^{-\imath 3q}|}e^{-\imath q(j-l)}\frac{dq}{2\pi} - \frac{2}{N}, \tag{32}$$

Without the second term, that stems from frustration, we would be able to apply strong Szegő limit theorem[?,?] to find the asymptotics of the Toeplitz determinant, and, therefore, of the spin-correlation functions. The second term is a correction, which can be understood as resulting from the part proportional to the delta function $\delta(q)$ in the symbol of the Toeplitz matrix $\mathbf{C}$. The asymptotics of such determinants has been studied in ref.[?]. The correction to the elements of the Toeplitz matrix results in a multiplicative correction to the determinant. Using Theorem 1 from there, in combination with the strong Szegő limit theorem, we get

$$\langle\sigma_1^x\sigma_{1+r}^x\rangle_g \overset{r\to\infty}{\simeq} (-1)^r(1-\tan^2\phi)^{3/4}\left(1-\frac{2r}{N}\right). \tag{33}$$

For a three-fold degenerate ground state when N is divisible by 3 the calculation is more complicated. Then we use directly (30) and resort to the numerical evaluation of pfaffians. However, we find that the result is the same, given by (33).

1.4 Expectation value of the String operator

For completeness we also compute the ground state expectation value of the string operator

$$O(r) = \sigma_1^y \sigma_2^x \left(\bigotimes_{j=3}^{r} \sigma_j^z \right) \sigma_{r+1}^x \sigma_{r+2}^y. \tag{34}$$

In terms of Majorana fermions (11) the operator reads

$$O(r) = \prod_{j=1}^{r} (-\imath A_j B_{j+2}). \tag{35}$$

Let us focus on the region $\phi \in (\pi/4, 3\pi/4)$. Since the correlators $\langle A_j A_l \rangle_g$ and $\langle B_j B_l \rangle_g$ vanish for $j \neq l$, the expectation value of the string operator can be expressed, using Wick theorem, as a determinant

$$\langle O(r) \rangle_g = \det \mathbf{D}, \tag{36}$$

where $\mathbf{D}$ is an $r \times r$ correlation matrix with the elements

$$\mathbf{D}_{j,l} = -\imath \langle A_j B_{l+2} \rangle_g \overset{N\to\infty}{\simeq} \int_0^{2\pi} \frac{1+\cot\phi \; e^{\imath 3q}}{|1+\cot\phi \; e^{\imath 3q}|} e^{-\imath q(j-l)} \frac{dq}{2\pi}. \tag{37}$$

For $\phi \in (-3\pi/4, -\pi/4)$ the only difference is that there is an additional factor $(-1)^r$ in front of the determinant in (36), because in this case $\sin\phi < 0$ in (13). The asymptotic behavior as $r \to \infty$ of the Toeplitz determinant $\det \mathbf{D}$ is obtained using the Strong Szegő limit theorem[?, ?]. The result is

$$\langle O(r) \rangle_g \overset{r\to\infty}{\simeq} \begin{cases} (1-\cot^2\phi)^{\frac{3}{4}}, & \phi \in (\frac{\pi}{4}, \frac{3\pi}{4}) \\ (-1)^r (1-\cot^2\phi)^{\frac{3}{4}}, & \phi \in (-\frac{3\pi}{4}, -\frac{\pi}{4}) \end{cases} \tag{38}$$

2 Kitaev chain

The diagonalization of the Kitaev chain Hamiltonian

$$H = -\mu \sum_{j=1}^{N} \left(c_j^\dagger c_j - \frac{1}{2} \right) - \sum_{j=1}^{N} \left[w\,(c_j^\dagger c_{j+1} + \text{h.c.}) - \Delta\,(c_j c_{j+1} + \text{h.c.}) \right] \tag{39}$$

with periodic BC is very similar to the diagonalization of H^- of the Cluster-Ising chain, discussed in section 1.1. The Hamiltonian is brought to a form of free fermions

$$H = \sum_{q\in\Gamma^-} \varepsilon_q \left(a_q^\dagger a_q - \frac{1}{2} \right), \tag{40}$$

where a_q are, again, Bogoliubov fermions, and the dispersion is now given by

$$\varepsilon_q = \sqrt{(4w\cos q + \mu)^2 + 4\Delta^2 \sin^2 q}, \quad q \neq 0, \pi \tag{41}$$

$$\varepsilon_0 = -2w - \mu, \tag{42}$$

$$\varepsilon_\pi = 2w - \mu. \tag{43}$$

The Bogoliubov angle satisfies

$$\tan\theta_q = -\frac{|2w\cos q + \mu + 2\Delta \sin q| + 2w\cos q + \mu}{2\Delta \sin q} \tag{44}$$

and

$$e^{\imath 2\theta_q} = -\frac{2w\cos q + \mu + 2\Delta\imath \sin q}{|2w\cos q + \mu + 2\Delta\imath \sin q|} \tag{45}$$

for $q \neq 0, \pi$. Note that the mode $q = \pi$ does not exist with FBC, since N is odd and momenta are quantized as integers.

Since in the Kitaev chain we do not have parity restrictions like in (4), the ground state can always be written as a state annihilated by all positive energy fermions d_q, defined in section (1.2). This also implies that the Majorana correlation functions in the ground state are given by (24) and (25), with $e^{\imath 2\theta_q}$ given by (45). This is valid both for N odd and N even.
